# Assessing the role of exogenous NO on plants and microbial communities in soil

**DOI:** 10.1093/ismeco/ycaf237

**Published:** 2025-12-16

**Authors:** Eduardo Pérez-Valera, Logapragasan Subramaniam, Pauline Trapet, Antoine Berger, Marie-Christine Breuil, Florian Engelsberger, Nicolas Brüggemann, Klaus Butterbach-Bahl, Michael Dannenmann, David Wendehenne, Laurent Philippot

**Affiliations:** Univ Bourgogne Europe, INRAE, Institut Agro Dijon, Agroécologie, 17 Rue Sully, Dijon 21000, France; Division “Terrestrial Bio-Geo-Chemistry”, Karlsruhe Institute of Technology (KIT), Institute for Meteorology and Climate Research Atmospheric Environmental Research (IMK-IFU), Kreuzeckbahnstr. 19, Garmisch-Partenkirchen 82467, Germany; Univ Bourgogne Europe, INRAE, Institut Agro Dijon, Agroécologie, 17 Rue Sully, Dijon 21000, France; Univ Bourgogne Europe, INRAE, Institut Agro Dijon, Agroécologie, 17 Rue Sully, Dijon 21000, France; Univ Bourgogne Europe, INRAE, Institut Agro Dijon, Agroécologie, 17 Rue Sully, Dijon 21000, France; Division “Terrestrial Bio-Geo-Chemistry”, Karlsruhe Institute of Technology (KIT), Institute for Meteorology and Climate Research Atmospheric Environmental Research (IMK-IFU), Kreuzeckbahnstr. 19, Garmisch-Partenkirchen 82467, Germany; Agrosphere (IBG-3), Forschungszentrum Jülich GmbH, Institute of Bio-and Geosciences, Jülich 52428, Germany; Division “Terrestrial Bio-Geo-Chemistry”, Karlsruhe Institute of Technology (KIT), Institute for Meteorology and Climate Research Atmospheric Environmental Research (IMK-IFU), Kreuzeckbahnstr. 19, Garmisch-Partenkirchen 82467, Germany; Center for Landscape Research in Sustainable Agricultural Futures - Land-CRAFT, Department of Agroecology, Aarhus University, Ole Worms Allé 3, Aarhus C 8000, Denmark; Division “Terrestrial Bio-Geo-Chemistry”, Karlsruhe Institute of Technology (KIT), Institute for Meteorology and Climate Research Atmospheric Environmental Research (IMK-IFU), Kreuzeckbahnstr. 19, Garmisch-Partenkirchen 82467, Germany; Univ Bourgogne Europe, INRAE, Institut Agro Dijon, Agroécologie, 17 Rue Sully, Dijon 21000, France; Univ Bourgogne Europe, INRAE, Institut Agro Dijon, Agroécologie, 17 Rue Sully, Dijon 21000, France

**Keywords:** nitric oxide, rhizosphere, *Arabidopsis thaliana*, *Solanum lycopersicum*, N_2_O, CO_2_, nitrogen cycling, bacteria, fungi

## Abstract

Nitric oxide (NO) is a reactive gas that functions as a signaling molecule regulating plant growth and stress responses, while also exerting various roles for microorganisms. In soil, NO is produced through microbial activity, plant metabolism, and physico-chemical processes. However, the impact of exogenous NO on plant physiology and the associated root microbiota remains unexplored. Here, we evaluated the effects of NO exposure on plant physiology, trace gas fluxes and N cycling, as well as the abundance, diversity, and composition of root-associated microbiota. We conducted two 37-day experiments with either *Arabidopsis thaliana* or tomato (*Solanum lycopersicum*) plants using innovative plant–soil mesocosms that allowed NO flushing while monitoring the CO_2_, N_2_O and NO fluxes. The mesocosms were subjected to four NO flushing periods (3–4 days each) at 0 ppbv or 400 ppbv. Our results revealed that exogenous NO_400_ exerted plant-specific effects. While flushing with NO_400_ had no effect on tomato plants or associated microbiota, it increased leaf area in Arabidopsis and modulated the expression of two genes involved in plant growth-defense balance compared to flushing with NO_0_. These changes in Arabidopsis physiology were concomitant with modest alterations in the fungal community and a decrease in the abundance of bacterial ammonia-oxidizers, ^15^N recovery as NO₃^−^, and cumulative CO₂ fluxes. However, it is still unclear how much of these effects were indirectly driven by plant–soil feedbacks. Our findings offer intriguing insights into the possible, though modest, effects of exogenous NO in shaping plant–microbe interactions.

## Introduction

Nitric oxide (NO) is a reactive trace gas which plays a critical role in tropospheric chemistry by affecting ozone and aerosol formation, as well as acid deposition. It originates from fossil fuel combustion, biomass burning, lightning, emissions from soils and other biogenic activity [1–3]. In the soil atmosphere, NO is primarily produced through both physico-chemical reactions and microbial processes [4, 5], although it can also be generated by plants [6]. Microorganisms involved in the nitrification and denitrification processes are the main biogenic contributors to NO production [7]. Previous studies showed that NO emitted from soil originates primarily from the uppermost layer of the soil profile, with NO concentrations in 5 cm depth averaging up to 400 ppbv on a monthly timescale in temperate forest soils, while declining in deeper soil layers [8]. Similar NO concentrations in the upper soil layer of up to 460 ppbv have also been reported for rainforest soils, where they were strongly influenced by soil temperature and moisture [9]. Yet, little is known about the biological importance of NO in the soil atmosphere for soil organisms and their interactions [10].

NO plays crucial roles in microorganisms, serving as a signaling molecule that regulates several bacterial metabolic pathways [11]. For example, it has been demonstrated that NO interacts with several bacterial transcriptional regulators involved in the response to oxidative stress, as well as in N-cycling processes such as nitrogen fixation, denitrification, and N_2_O reduction [11]. Moreover, NO plays a role in protection against antibiotics [12], formation of biofilms and motility [1, 13]. In fungi, NO contributes to plant infection processes, and also regulates fungal morphogenesis and reproduction [14]. At high concentrations, NO induces both nitrosative and oxidative damages, resulting in various toxic effects on bacteria and fungi. These effects are strain-dependent and include the direct modification of membrane proteins, lipid peroxidation, and DNA cleavage [14, 15]. However, the importance of NO has most often been studied in vitro at the population level and, therefore, the response of microbial communities to NO in complex environments, such as soil and plant–soil systems, remains unexplored due to methodological constraints in both dynamically manipulating and measuring trace gas fluxes [16].

In plants, NO is also a key signaling molecule activating defense mechanisms following pathogen attack [17]. Its effectiveness in protecting plants against microbial pathogens as an antimicrobial agent, due to its toxic effects, depends however on its concentration and the plant’s physiological conditions—whether under stress or not [18]. At low concentrations, NO can also influence seed germination, root development, stomatal closure, and adaptive responses to biotic and abiotic stresses, whereas excessive levels suppress leaf expansion [19–23]. Although NO is central to several physiological and biochemical processes in plants, little is known about the ability of plants to perceive exogenous NO and its consequence for plant physiology. Studies based on the exposure of plant tissues to chemical NO donors or of the aerial part of plants to gaseous NO have shown that such treatments modulate the expression of numerous genes, promote NO-dependent post-translational protein modifications, and affect plant morphological traits and physiological processes [24]. Yet, the relevance of NO concentrations typically found in the soil atmosphere for plant physiology, growth and plant-microbe interactions remains unclear. This is partly due to the lack of reliable experimental systems that enable the precise manipulation and quantification of trace gas concentrations, such as NO, in soil.

Here, we investigated the extent to which exogenous NO may affect directly or indirectly plant physiology and growth as well as soil and plant-associated microbial communities, with a focus on C and N cycling processes in soil. For this purpose, we used a newly developed innovative plant–soil mesocosm system that dynamically changes background NO concentrations while also allowing the soil-atmosphere exchange of other trace gases to be measured [25]. Arabidopsis and tomato plants were grown under 0 ppbv or 400 ppbv NO for 5 weeks before analyzing the bacterial and fungal communities in soil, rhizosphere and roots, plant physiological status, N balance as well as CO_2_, N_2_O and NO fluxes. We hypothesized that (i) due to its antimicrobial properties, exogenous NO would detrimentally affect the abundance and diversity of microbial communities, (ii) N-cycling microbial communities would particularly be affected, as NO can also act both as a substrate and a regulatory molecule, (iii) root-associated microbes would be more susceptible due to direct and indirect effects mediated by exogenous NO-induced physiological and morphological changes in Arabidopsis and tomato plants.

## Materials and methods

### Plant and soil materials, plant germination, and mesocosm preparation

The soil was collected at the CEREEP research station, France (N 48°17′14.48″, E 2°40′34.64″). The soil is classified as sandy loam (clay: 6.9%; silt: 19.0%; sand: 74.1%) with a C/N ratio of 12.25 and a pH (H_2_O) of 5.22. Soil mesocosms were prepared by filling cylindrical Plexiglas cuvettes (126.5 mm diameter, 200 mm height) with 1633 g of sieved and homogenized soil corresponding to a depth of 10 cm [25]. Water was added to reach 20% WFPS on the first day, and then, after the transfer of the seedlings, it was maintained at 40% WFPS throughout the experiment.

Arabidopsis and tomato seeds were first surface-sterilized with 70% ethanol (1 min) and 6% sodium hypochlorite (10 min) and then rinsed five times with sterile distilled water. Two independent experiments were carried out by sowing either three *A. thaliana* Col-0 (WT) or three tomato *S. lycopersicum* (Moneymaker) seedlings per mesocosm.

### Automated plant–soil mesocosm system and flux measurements

Twelve mesocosms per plant species were incubated for 37 days at 20°C in the automated plant–soil mesocosm system (AU-MES) designed for controlled headspace and soil flushing with nitric oxide (NO), as detailed in [25]. Briefly, these mesocosms have an integrated LED lighting system in the upper lid for optimum plant growth with a 10- h light cycle and a soil flushing inlet at the bottom lid to ensure even soil flushing. Six mesocosms were soil-flushed with NO at 400 ppbv (NO_400_) on specific days (7–11, 16–18, 23–26, and 32–35), while the remaining six were soil-flushed the same days with ambient air (NO_0_) and served as controls (Fig. S1). On day 13, all mesocosms were fertilized with (^15^NH_4_)_2_SO_4_ at a 30% and 70% atom enrichment for the Arabidopsis and tomato experiments, respectively, and applied at a rate of 60 kg N ha^−1^. A NO concentration of 400 ppbv was selected as representative of the range of values reported in a previous year-long study, in which monthly averages of up to 400 ppbv were observed in rainforest soils [8]. This concentration is also consistent with NO levels of up to 460 ppbv measured at a tropical forest site during a one-month monitoring period [9].

The gas fluxes from the mesocosms were routed through multiposition valves to a multigas analyzer, enabling continuous monitoring of trace gas concentrations (CO_2,_ N_2_O, and NO) via mid-infrared laser spectrometry. Measurements followed a 144-min sampling sequence, with each mesocosm (outlet) and the reference chamber (inlet) being measured for 6 min across the 12 mesocosms. The system operated in two modes: NO soil flushing mode and headspace gas flux measurement mode (Fig. S1). Throughout the incubation, alternating periods of soil NO flushing and trace gas flux measurements were conducted.

### Sampling of the mesocosms

After 37 days, soil samples (hereinafter referred to as “bulk soil”) were collected in triplicate from each mesocosm. Soil samples were collected and separated into two depths (0–5 cm and 5–10 cm), taking into account the potential effects of moistening and ^15^N fertilization, which was applied to the top layer. The samples were then pooled and homogenized by layer for each mesocosm, resulting in six replicates per depth and NO concentration. To collect the rhizospheric soil, the loose soil was first gently removed by kneading and shaking the roots. The roots from all three plants within each mesocosm were combined in a clean, sterile 15 ml Falcon tube containing 2.5 ml phosphate buffer (per liter: 6.33 g of NaH_2_PO_4_, 16.5 g of Na_2_HPO_4_, 200 μl Silwet L-77). The rhizospheric soil was separated from the roots by vortexing at maximum speed for 15 s at 3200 × g. Most of the supernatant was removed and the remaining loose pellets were resuspended and transferred to a 1.5 ml tube, then centrifuged for 5 min at 10 000 × g. The resulting pellet was designated as the rhizosphere compartment. For the endophytic compartment, the cleaned roots from the previous vortexing step were transferred to another 15 ml Falcon tube containing 2.5 ml phosphate buffer and sonicated for less than 5 min to avoid overheating. After removing the buffer, sonicated roots were defined as the endophytic compartment. All bulk soil, rhizosphere, and root endophytic samples were transferred into 2 ml tubes and stored at −80°C.

### Plant RNA extraction and RT-qPCR relative gene expression

RNA samples were isolated from one frozen leaf, first tissue was ground with a potter pestle, and RNA was extracted using the SV Total RNA isolation system as per the manufacturer’s guidelines (Promega). The quality of the RNA was verified prior to reverse transcription with High-Capacity cDNA Reverse Transcriptase Kit (Applied Biosystems). Reverse transcription quantitative PCR was carried out following the protocol provided by the manufacturer using a Go-Taq qPCR master mix kit (Promega). Cq and primer efficiency for each well were obtained using LinReg software. Gene expression level was then calculated following the method described by Ganger *et al.* [26]. The ΔΔCq values were used for statistical analyses, and the relative gene expression (10^−ΔΔCq^) was plotted. We quantified the gene expression involved in iron homeostasis, defense-growth balance and nitrogen metabolism in Arabidopsis and tomato plants (Table S1). Gene expression levels were normalized to two reference genes for Arabidopsis—*At4g26410* and *AtPTB*— [27] and three reference genes for tomato—*SlActin* [28], *SlTIP41* and *Slg025390.2* [29]. The reverse transcription reactions were done with technical duplicates using the primers detailed in Table S2.

### Plant traits

To avoid stressing plants for biochemical analyses, the leaf area (in cm^2^) was quantified using images of the mesocosms taken from above on day 37 (sampling day). Plants showing severe growth limitations were excluded from the analysis. In the case of the tomato experiment, it was not possible to quantify the leaf area due to the overlapping leaves. Only tomato plants were used for biomass and ^15^N analyses, as the limited plant material available for Arabidopsis prevented further analyses.

### Assessment of microbial community composition and diversity

DNA was extracted from bulk soil (2 NO treatments × 2 soil depths × 6 replicates) as well as rhizospheric soil samples (2 NO treatments × 6 replicates) from both Arabidopsis and tomato using the DNeasy PowerSoil-htp 96 well DNA isolation kit (Qiagen, France). For the root samples (2 NO treatments × 6 replicates), the DNA was extracted using the DNeasy Plant kit (Qiagen, France) according to the manufacturer’s instructions. Extracted DNA was quantified using the Quant-IT dsDNA HS Assay Kit (Invitrogen, Carlsbad, CA, USA). The V3-V4 16S rRNA gene region was amplified using a 2-step PCR as described in [30]. PCR products were verified on a 2% agarose gel and normalized using the SequalPrep Normalisation plate kit (Invitrogen, Carlsbad, CA, USA). Sequencing was performed on an Illumina MiSeq (2 × 250 bp) using the MiSeq Reagent Kit v2.

Demultiplexing and trimming of Illumina adaptors and barcodes were performed using Illumina MiSeq Reporter software (version 2.5.1.3). Sequence data for both 16S rRNA and ITS were analyzed using an in-house Python pipeline (available upon request), as described in [30]. Briefly, paired-end sequences were merged, and short sequences (<400 bp for 16S and <300 bp for ITS) and those identified as chimeras removed. OTU identity thresholds were set at 94% for 16S and 97% for ITS. Taxonomy was assigned using UCLUST (USEARCH v11) [31] and the SILVA database (v138.1/2020) [32] for 16S rRNA and using BLAST [33] and the UNITE reference database (v8.3/2021) [34] for ITS. Sequences from the 16S rRNA gene that were classified as mitochondria or chloroplast were excluded. A total of 8163 OTUs (50 713 ± 20 039 (mean ± SD) reads per sample) and 6292 OTUs (59 946 ± 7774 reads per sample) were generated for the 16S rRNA and ITS, respectively.

### Quantification of total bacterial, ammonia-oxidizing and denitrifier communities

Real-time quantitative PCR assays were used to determine the abundances of total bacterial, fungal, ammonia-oxidizing and denitrifying communities in both bulk soil and rhizosphere samples only, due to low DNA yields from the root samples. Total bacterial community and fungal abundances were quantified using 16S rRNA and ITS primers previously described [35]. Ammonia-oxidizing archaeal (AOA), bacterial (AOB) and comammox communities were quantified as in [36]. Denitrifying communities carrying the *nirK* and *nirS* genes were assessed as in [30] and *nosZI* and *nosZII* genes as in [37]. Negative controls were included in each measurement and inhibition tests prior to qPCR were negative.

### Soil and plant N-pool analyses

The ^15^N balance was assessed in bulk soil samples from 0–5 cm and 5–10 cm depth, focusing on ^15^N recovery in total nitrogen (TN), extractable organic and mineral N, and microbial biomass N (MBN). Homogenized samples (50 g) were extracted with 0.5 M K_2_SO_4_ (1:2, soil:solution) [38] and stored at −20°C. NH_4_^+^ and NO_3_^−^ concentrations were measured by microplate spectrophotometry [39]. Total dissolved N (TDN) and dissolved organic carbon (DOC) were analyzed using a TOC/TN analyzer. Dissolved organic N (DON) was calculated as the difference between TDN and mineral N [40]. MBN was determined by chloroform fumigation extraction without a correction factor [39]. ^15^N enrichment in NH_4_^+^, NO_3_^−^, DON, and MBN was quantified by sequential diffusion in acid traps followed by elemental analysis–isotope ratio mass spectrometry (EA-IRMS) [40].

The tomato plants were separated into above-ground (leaves and shoots) and below-ground (roots) compartments. The roots were rinsed with tap water, and all plant samples were dried at 55°C until a constant weight was reached, then finely ground and stored in tin capsules over silica gel for ^15^N analysis. In addition, ~10 g of soil from each depth was similarly processed to assess ^15^N recovery. ^15^N enrichment and total N concentrations in both plant sections and soil were quantified using EA-IRMS. Isotopic data and ^15^N tracer recovery across plant and soil nitrogen pools were calculated using established formulas [40].

### Statistical analysis

Statistical analyses were performed using R statistical software (version 4.2.2) [41]. Differences in plant gene expression and leaf area were evaluated using t-tests with log10-transformed data. Taxonomic bar plots from microbial communities were computed using microeco (v1.8.0) [42]. Alpha diversity (i.e. OTU richness and Shannon diversity index) was calculated after rarefaction (13 000 and 15 700 reads per sample without replacement for 16S rRNA and ITS, respectively). Samples under rarefaction limits were excluded. Principal Coordinates Analysis (PCoA) and permutational multivariate analysis of variance (PERMANOVA) were run using Bray–Curtis dissimilarity matrices. Beta diversity PCoA analyses were plotted using phyloseq v1.41.1 [43] using rarefied tables. PERMANOVAs were run on the rarefied matrices using the adonis2 function (999 permutations) in vegan v2.6–4 [44]. Pairwise differences in bacterial and fungal composition were analyzed using the pairwise.adonis function (999 permutations, corrected *P* < .05 using Benjamini-Hochberg) of the pairwiseAdonis v0.4.1 package [45]. In bulk soil samples, the effects of NO treatment, soil depth, and their interaction on alpha diversities, as well as on the abundance of bacteria, fungi, ammonia oxidizers, denitrifiers, and nitrogen pools, were assessed using ANOVA. Additionally, similar ANOVAs were conducted across all sample types (bulk, rhizosphere, and root) to evaluate the effects of NO treatment, compartment, and their interaction. ANOVAs were run independently for Arabidopsis and tomato experiments. Pairwise differences in alpha diversity, microbial abundances and N pools were evaluated using non-parametric Kruskal-Wallis tests followed by Fisher’s least significant difference with Benjamini-Hochberg correction (*P <* .05).

Flux data were preprocessed using a custom R-script, focusing on the complete time series and individual phases, with trace gas fluxes calculated only during headspace flushing periods [25]. T-tests (*P* = .05) were applied to evaluate NO effects on leaf area, soil-atmosphere trace gas fluxes and compare cumulative fluxes between control and NO-treated soils. Data visualization, including gas flux and nitrogen dynamics graphs, was conducted with OriginPro 2020b (OriginLab Corporation).

## Results

### Plant responses to NO_0_ and NO_400_ treatments

**Figure 1:**
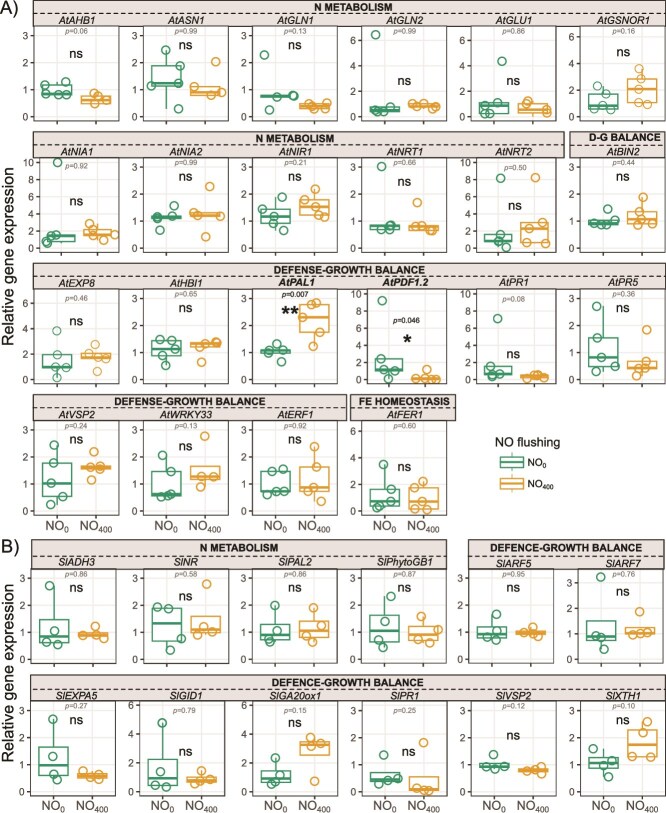
Relative expression of plant genes involved in N metabolism, defense-growth balance and iron homeostasis in the leaves of (A) Arabidopsis and (B) tomato plants subjected to NO treatments (NO_0_ and NO_400_). For each box, the central horizontal line represents the median; the lower and upper edges correspond to the first and third quartiles, respectively; whiskers extend to the minimum and maximum values no further than 1.5 times the interquartile range. Asterisks indicate statistical differences (t-tests: ns *not significant*).

Enrichment of the ambient air stream with NO at 400 ppbv in the automated plant–soil mesocosms had a limited impact on the transcript levels of plant genes related to N metabolism, defense mechanisms, hormone responses and growth (Fig. 1). Thus, only the transcripts levels of *AtPAL1* and *AtPDF1.2*—involved in plant metabolism and/or the plant defensive response – were significantly affected by NO_400_ in Arabidopsis, showing an increase of 112% and a decrease of 89.56% compared to NO_0_, respectively. NO_400_ also led to a significantly larger leaf area in Arabidopsis plants, with an average increase of 61% compared to NO_0_ (Fig. S2). A small but significant increase in the tomato belowground, but not aboveground, biomass was also observed in response to NO_400_ without any significant changes in the plant N-content (Table S3).

### Taxonomic composition of bacterial and fungal communities in response to increased NO

Increased NO concentration in the soil atmosphere did not alter the relative abundance of the major bacterial or fungal phyla, regardless of the compartment or of the plant species (Fig. S3). The bacterial communities in bulk soil, both at 0–5 cm and 5–10 cm depth, and in the rhizosphere of Arabidopsis and tomato plants, were dominated by Firmicutes (35%–49% relative abundance), followed by Gammaproteobacteria (10%–15%), Actinobacteria (9%–13%) and Alphaproteobacteria (6%–10%) (Fig. S3A). At the genus level, *Bacillus* (11%–23%) and *Tumebacillus* (2%–10%) dominated in bulk soil (Fig. S4A). The predominant bacterial groups in plant roots strongly differed from those in the rhizosphere or bulk soil, with Gammaproteobacteria—*Massilia* in Arabidopsis and *Pseudomonas* in tomato—dominating in the roots for both plant species, followed by Alphaproteobacteria and Bacteroidetes. In fungal communities, Ascomycota was the dominant phyla in the bulk soil at both depths and in the rhizosphere of tomato plants, with *Pseudeurotium* as the more abundant genus (Figs. S3B and S4B). Basidiomycota, mainly the genus *Sebacina,* dominated in Arabidopsis roots (>93%) whereas the composition of the fungal community was more even in tomato roots with similar proportions of Basidiomycota, Ascomycota, Olpidiomycota and Glomeromycota.

### Effects of NO on microbial alpha diversity

**Figure 2:**
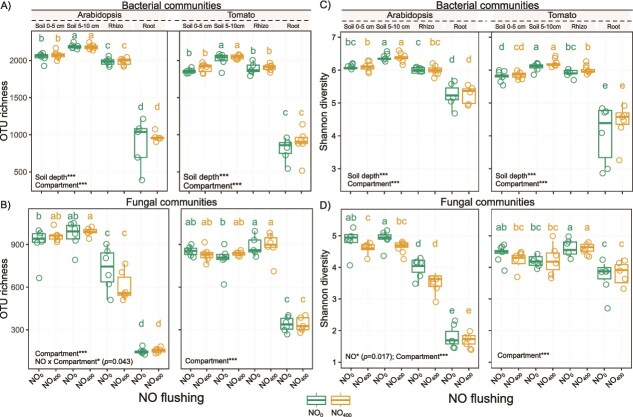
Boxplots showing the differences in the observed OTU richness of (A) bacterial and (B) fungal communities, and the Shannon diversity of (C) bacterial and (D) fungal communities in bulk soil, rhizosphere and root samples of Arabidopsis and tomato subjected to NO treatments (NO_0_ and NO_400_). For each box, the central horizontal line indicates the median, while the lower and upper edges represent the first and third quartiles, respectively. The whiskers extend to the minimum and maximum values, provided they do not exceed 1.5 times the interquartile range. Distinct letters above the boxes indicate significant differences based on non-parametric Kruskal-Wallis tests, followed by Fisher’s least significant difference with Benjamini-Hochberg correction for pairwise comparisons (*P <* .05). Statistical significance for the effects of (i) NO, soil depth, and their interaction, and (ii) NO, compartment (bulk soil, rhizosphere and root), and their interaction, is indicated by asterisks in the figure (^*^P < .05, ^**^P < .01, ^***^P < .001), with ANOVA model outputs provided in Table S4.

Exogenous NO had no effect on the alpha diversity of the bacterial communities, whereas it significantly altered the fungal diversity (Shannon index) in the Arabidopsis experiment (Fig. 2). A significant NO × compartment interaction was also found for fungal richness in the Arabidopsis experiment (Fig. 2B, Table S4). Nevertheless, differences in the alpha diversity were more pronounced based on the soil depth, plant species and plant compartment. Thus, the bacterial and fungal OTU richness were primarily affected by the compartment, with decreases ranging between 54 and 58% in the roots compared to the other compartments, regardless of the plant species. Similarly, the Shannon index was the lowest in the roots but without significant differences with the bulk soil for fungi in tomato (Fig. 2C and D). For both bacterial and fungal communities, the rhizosphere effect was stronger in Arabidopsis than in tomato. Finally, the soil depth had a significant effect on the bacterial OTU richness and the Shannon index, with higher diversity in the bulk soil at 5–10 cm depth compared to 0–5 cm depth (Fig. 2A, C, Table S4).

### Beta diversity of microbial communities

**Figure 3:**
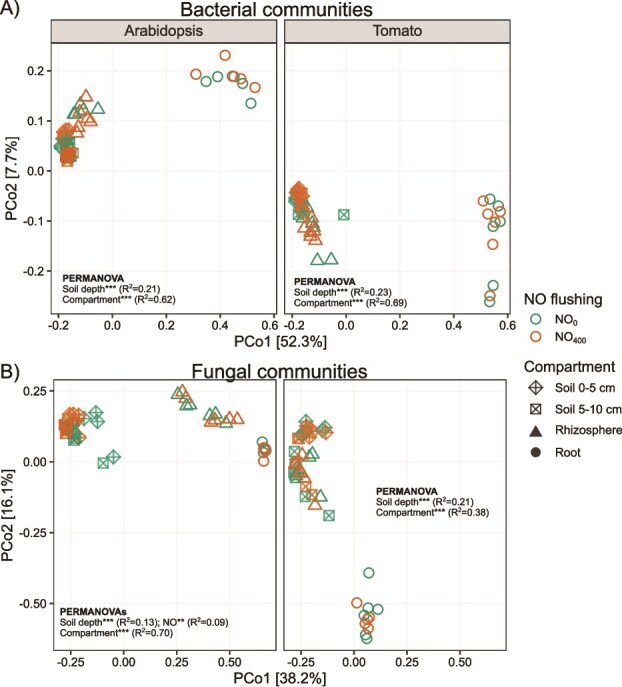
Principal Coordinate Analysis (PCoA) of Bray–Curtis dissimilarities of (A) bacterial communities and (B) fungal communities in bulk soil, rhizosphere and root samples of Arabidopsis and tomato subjected to NO treatments (NO_0_ and NO_400_). Two PERMANOVAs per plant species and microbial group were performed to assess (i) the effects of NO, soil depth, and their interaction by only including bulk soil samples, and (ii) the effects of NO, compartment (soil, rhizosphere, and root), and their interaction. Statistical significance levels are indicated by asterisks in the figure (^*^P < .05, ^**^P < .01, ^***^P < .001).

Next, we tested the effect of exogenous NO concentration on the beta diversity of bacterial and fungal communities using principal coordinate analysis (PCoA) and permutational multivariate analysis of variance (PERMANOVA) (Fig. 3, Table S5). In the Arabidopsis experiment, NO_400_ had a limited but significant impact on the specific composition of fungal communities in the bulk soil at both depths, as shown by the PERMANOVA (Fig. 3) and the pairwise PERMANOVAs (Table S5). Nevertheless, the primary drivers of both microbial communities were the compartment followed by soil depth (Fig. 3).

### Abundance of total bacterial and fungal communities as well as of ammonia-oxidizers and denitrifiers

**Figure 4:**
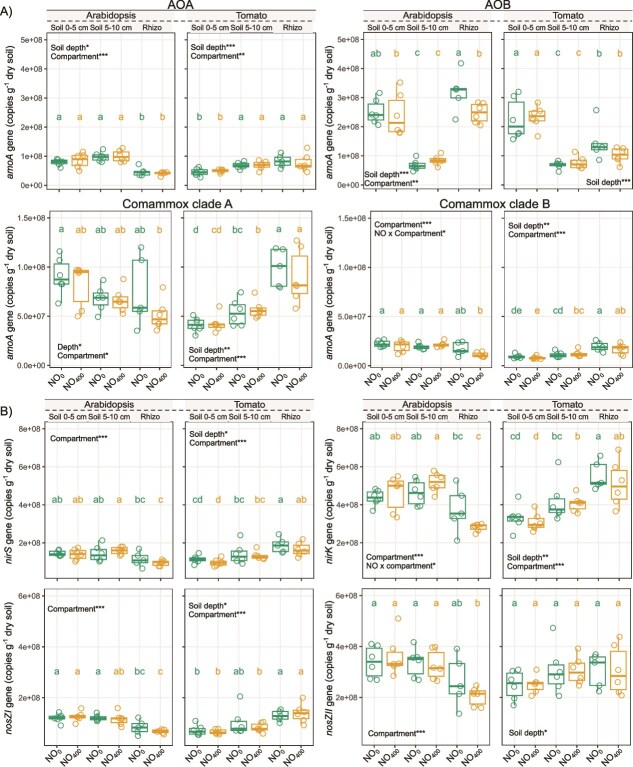
Boxplots showing the abundance of (A) ammonia oxidizers based on *amoA* gene copies of AOA, AOB, comammox clade A and comammox clade B, and the abundance of (B) denitrifiers based on *nirS*, *nirK*, *nosZI,* and *nosZII* gene copies, in bulk soil and the rhizosphere of Arabidopsis or tomato subjected to NO treatments (NO_0_ and NO_400_). For each box, the central horizontal line indicates the median, while the lower and upper edges represent the first and third quartiles, respectively. The whiskers extend to the minimum and maximum values, provided they do not exceed 1.5 times the interquartile range. Statistical tests are run independently for each experiment (Arabidopsis or tomato) and gene. Distinct letters above the boxes indicate significant differences based on non-parametric Kruskal-Wallis tests, followed by Fisher’s least significant difference with Benjamini-Hochberg correction for pairwise comparisons (*P <* .05). Statistical significance for the effects of (i) NO, soil depth, and their interaction, and (ii) NO, compartment (bulk soil, rhizosphere and root), and their interaction, is indicated by asterisks in the figure (^*^P < .05, ^**^P < .01, ^***^P < .001), with ANOVA model outputs provided in Table S4.

Quantification of ammonia-oxidizing and denitrifier community abundances by qPCR revealed that NO_400_ had a significant effect on ammonia oxidizers and denitrifiers only in the Arabidopsis experiment (Fig. 4, Table S4), with NO_400_ leading to a significant decrease in the abundance of ammonia-oxidizing AOB compared to NO_0_. A significant interaction was also observed between NO treatment and compartment (i.e. bulk soil or Arabidopsis rhizosphere) for the abundance of ammonia-oxidizing comammox clade B and *nirK*-denitrifiers. Ammonia-oxidizer and denitrifier abundances also varied significantly with soil depth, compartment and plant species. Total bacterial and fungal abundances were unaffected by NO in either species (Fig. S5, Table  S4).

### Emissions and cumulative fluxes of CO_2_, N_2_O, and NO

**Figure 5:**
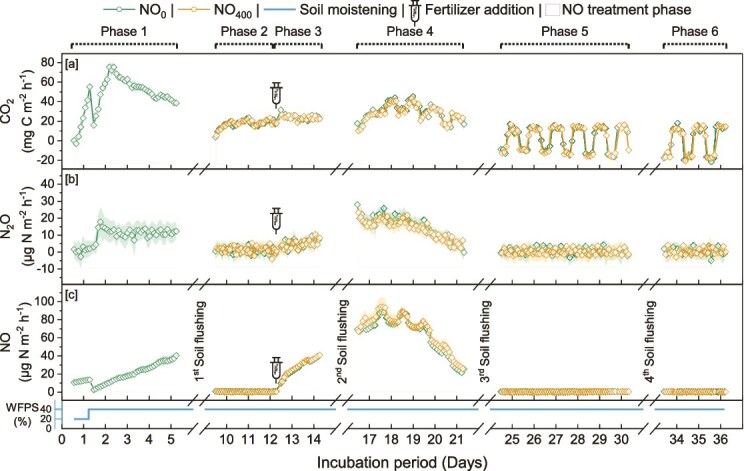
Soil fluxes of CO_2_, N_2_O and NO from the Arabidopsis plant–soil experiment subjected to NO treatments (NO_0_ and NO_400_) over a 37-day incubation. Treatment phases, including ambient air, elevated NO (400 ppbv-NO), soil moistening, and fertilizer additions (using (^15^NH_4_)_2_SO_4_), are marked at the top, with NO treatment periods shaded in yellow. Intervals of interrupted surface flux measurements due to bottom-soil flushing, are indicated by dotted sections. Phases (P1-P6) are labelled to correspond with cumulative emissions reported in Table 1. Data are presented as mean ± standard error (SE) across six replicates (N = 6).

**Figure 6:**
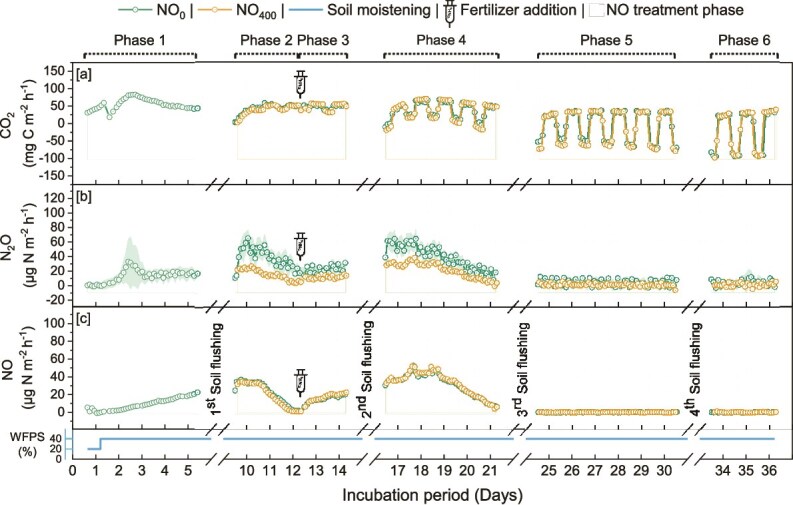
Soil fluxes of CO_2_, N_2_O and NO from the tomato plant–soil experiment subjected to NO treatments (NO_0_ and NO_400_) over a 37-day incubation. Treatment phases, including ambient air, elevated NO (400 ppbv-NO), soil moistening, and fertilizer additions (using (^15^NH_4_)_2_SO_4_), are marked at the top, with NO treatment periods shaded in yellow. Intervals of interrupted surface flux measurements due to bottom-soil flushing, are indicated by dotted sections. Phases (P1-P6) are labelled to correspond with cumulative emissions reported in Table 1. Data are presented as mean ± standard error (SE) across six replicates (N = 6).

Emissions of CO_2_, N_2_O and NO gases were monitored throughout the 37-day incubations periods in all mesocosms during six treatment phases (i.e. seedling transfer, ^15^N fertilization and 4 NO flushing periods; Figs. 5 and 6). Cumulative fluxes of CO_2_, N_2_O and NO varied significantly with the experiment, the treatment phase, and the light cycle (Table 1). After phase 3 (2^nd^ NO flushing) corresponding to the leaf emergence, the CO_2_ emissions showed a clear pattern aligned with the light cycles, with higher emissions when the lighting was off than when it was on. In the Arabidopsis experiment, NO_400_ significantly reduced CO_2_ emissions with lights on during phases 3 and 4, resulting in a significant decrease by 12% in cumulative CO_2_ emissions (Table 1). On the contrary, with lights off, NO_400_ significantly increased CO_2_ respiration during phases 2, 3 and 6. In the tomato experiment, similar effects of NO_400_ on CO_2_ fluxes were observed with decreases with light on (phases 2 and 4) and increases with light off (phase 4).

**Table 1:** Cumulative gas fluxes of carbon dioxide (CO_2_), nitrous oxide (N_2_O) and nitric oxide (NO) measured for both Arabidopsis and tomato experiments across the entire incubation period and individual phases (P1–P6). Data are presented as mean ± standard error (SE) based on six replicates, with statistically significant differences indicated by asterisks (^*^P < .05, ^**^P < .01).

**Gas species**	**Plant**	**Condition**	**Nitric oxide treatment**	**Phase 1** Days (0–6)	**Phase 2** Days (10–12.5)	**Phase 3** Days (12.5–15)	**Phase 4** Days (17–22)	**Phase 5** Days (25–31)	**Phase 6** Days (34–37)	∑**Phase 1–6** Days (0–37)
**CO**_**2**_ (mg C m^−2^)	Arabidopsis	Lights ON	NO_0_	2536 ± 199	515 ± 19	587 ± 12^**^	1447 ± 47^*^	−452 ± 28	−375 ± 23	4540 ± 412^*^
			NO_400_		474 ± 28	**476 ± 24**	**1269 ± 47**	−517 ± 85	−401 ± 66	**3556 ± 206**
		Lights OFF	NO_0_	3410 ± 179	701 ± 23^*^	568 ± 9^**^	2035 ± 46	841 ± 20	359 ± 7^**^	8147 ± 322
			NO_400_		**762 ± 18**	**630 ± 11**	2063 ± 34	803 ± 39	**402 ± 12**	7836 ± 137
		Total	NO_0_	5946 ± 244	1215 ± 21	1155 ± 19	3482 ± 69	389 ± 36	−16 ± 29	12 687 ± 384^**^
			NO_400_		1236 ± 15	1107 ± 26	3332 ± 64	286 ± 68	1 ± 66	**11 392 ± 218**
	Tomato	Lights ON	NO_0_	2190 ± 89	1091 ± 54^*^	927 ± 77	1064 ± 128^*^	−2933 ± 192	−2377 ± 118	−4 ± 446
			NO_400_		**943 ± 49**	871 ± 38	**604 ± 84**	−3398 ± 293	−1474 ± 612	−287 ± 824
		Lights OFF	NO_0_	3058 ± 89	1846 ± 59	1336 ± 33	3305 ± 111^*^	1956 ± 66	846 ± 103	12 220 ± 188
			NO_400_		1759 ± 54	1351 ± 37	3556 ± 79	1870 ± 109	710 ± 83	12 387 ± 236
		Total	NO_0_	5248 ± 107	2937 ± 55^*^	2263 ± 79	4369 ± 55	−977 ± 225	−1531 ± 221	12 216 ± 360
			NO_400_		2701 ± 90	2222 ± 66	4159 ± 125	−1528 ± 283	−764 ± 602	12 100 ± 959
**N**_**2**_ **O** (μg N m^−2^)	Arabidopsis	Lights ON	NO_0_	494 ± 36	34 ± 35	103 ± 16	952 ± 55^*^	4 ± 18	15 ± 20	1564 ± 116
			NO_400_		56 ± 18	85 ± 24	**825 ± 47**	23 ± 32	19 ± 28	1539 ± 103
		Lights OFF	NO_0_	619 ± 55	24 ± 16	132 ± 20	863 ± 64	47 ± 16	0 ± 5	1583 ± 146
			NO_400_		58 ± 13	143 ± 7	836 ± 31	−9 ± 41	10 ± 20	1758 ± 68
		Total	NO_0_	1113 ± 88	58 ± 33	235 ± 33	1815 ± 106^*^	50 ± 23	14 ± 21	3147 ± 249
			NO_400_		114 ± 24	228 ± 19	**1661 ± 46**	15 ± 69	29 ± 24	3297 ± 150
	Tomato	Lights ON	NO_0_	978 ± 422	1113 ± 270	466 ± 123^*^	2411 ± 416^*^	312 ± 64^**^	165 ± 86	5900 ± 1026^**^
			NO_400_		528 ± 16	**256 ± 25**	**1316 ± 43**	**113 ± 35**	21 ± 38	**2909 ± 362**
		Lights OFF	NO_0_	860 ± 137	1547 ± 486^*^	605 ± 142^*^	2295 ± 420^*^	290 ± 139	167 ± 121	5660 ± 942^**^
			NO_400_		566 ± 26	298 ± 17	**1253 ± 87**	99 ± 26	34 ± 48	**3180 ± 301**
		Total	NO_0_	1838 ± 495	2660 ± 752^*^	1072 ± 263	4706 ± 835^*^	622 ± 200^*^	332 ± 197	11 559 ± 1674^**^
			NO_400_		**1094 ± 39**	553 ± 24	**2570 ± 120**	201 ± 60	65 ± 90	**6089 ± 653**
**NO** (μg N m^−2^)	Arabidopsis	Lights ON	NO_0_	928 ± 78	22 ± 2	572 ± 14^**^	3906 ± 218	34 ± 1	13 ± 1	5565 ± 210
			NO_400_		23 ± 1	**489 ± 21**	3862 ± 364	36 ± 2	13 ± 2	5259 ± 547
		Lights OFF	NO_0_	1322 ± 85	21 ± 2^*^	737 ± 17^*^	3756 ± 192	30 ± 1	8 ± 2	5912 ± 210
			NO_400_		**26 ± 2**	**888 ± 62**	4181 ± 448	33 ± 2	9 ± 1	6420 ± 698
		Total	NO_0_	2250 ± 160	42 ± 4	1308 ± 31	7662 ± 396	64 ± 2	21 ± 2	11 477 ± 410
			NO_400_		49 ± 3	1377 ± 82	8042 ± 811	70 ± 4	22 ± 2	11 680 ± 1245
	Tomato	Lights ON	NO_0_	346 ± 41	778 ± 75	289 ± 17	1939 ± 62	37 ± 2	22 ± 1	3380 ± 116
			NO_400_		720 ± 68	340 ± 30	1892 ± 181	40 ± 2	117 ± 90	3473 ± 343
		Lights OFF	NO_0_	596 ± 60	777 ± 65	459 ± 19	1858 ± 75	35 ± 4	15 ± 1	3658 ± 47
			NO_400_		664 ± 59	462 ± 40	1988 ± 194	33 ± 4	299 ± 282	4097 ± 557
		Total	NO_0_	942 ± 95	1554 ± 118	748 ± 32	3797 ± 118	72 ± 6	36 ± 2	7038 ± 151
			NO_400_		1384 ± 125	801 ± 71	3880 ± 371	73 ± 6	416 ± 372	7570 ± 898

The impact of NO_400_ compared to NO_0_ was more pronounced on N_2_O emissions, particularly in the tomato experiment (Figs. 5 and 6). Thus, NO_400_ reduced N_2_O emissions by a total of 59% after the second NO flush (during phase 4) and by a total of 47% over the entire incubation period, regardless of light conditions (Table 1). In the Arabidopsis experiment, NO_400_ significantly decreased N_2_O emissions only during phase 2 with lights on.

NO emissions were significantly affected by flushing with NO_400_ only in the Arabidopsis experiment, increasing by 17 to 19% when lights were off in phase 2 and 3, while decreasing by 14% after fertilization in phase 3 with lights on.

### Nitrogen dynamics in response to exogenous NO

Exogenous NO significantly altered the ^15^N recovery of soil nitrogen forms compared to the 0 ppbv NO control, though the impact was limited and varied depending on the nitrogen form, the plant experiment, and the soil depth (Table 2). Thus, in the Arabidopsis experiment, NO_400_ led to a significant decrease in total ^15^N recovery for NO_3_^−^ while a higher recovery was observed for MBN but only at 0–5 cm. In the tomato experiment, NO_400_ resulted in a decreased ^15^N recovery for both NO_3_^−^ and total nitrogen (TN) at 0–5 cm and an increased ^15^N recovery at 5–10 cm for NO_3_^−^ only. Overall, higher ^15^N recoveries were consistently observed in the upper soil layer (0–5 cm), compared to the lower layer (5–10 cm), as well as in the tomato aboveground biomass (~80%) compared to the belowground biomass (~5%). However, exogenous NO had no significant impact on the ^15^N recovery between aboveground and belowground biomass (Table S6). In the Arabidopsis experiment only, exogenous NO consistently lowered NH_4_^+^ concentrations independent of soil depth, whereas NO_3_^−^ exhibited variable responses (Fig. S6, Table S4).

**Table 2:** Impact of exogenous NO on the N balance. ^15^N recovery percentages in both Arabidopsis and tomato experiments, showing nitrate (NO_3_^−^), dissolved organic nitrogen (DON), microbial biomass nitrogen (MBN) and total nitrogen (TN) across two soil depth segments (0–5 cm and 5–10 cm), as well as cumulative depth, under varying NO treatments. In the Arabidopsis experiment, the soil was labelled with (^15^NH_4_)_2_SO_4_ at a 30% atom ^15^N enrichment, while in the tomato experiment, the soil was labelled with (^15^NH_4_)_2_SO_4_ at a 70% atom ^15^N enrichment, and both were applied at a rate of 60 kg N ha^−1^. Values are reported as mean ± standard error (SE) from six replicates, with statistically significant differences denoted by asterisks (^*^P < .05).

	**Plants**	**Nitric oxide treatment**	^**15**^ **N Recovery % (0–5 cm)**	^**15**^ **N Recovery % (5–10 cm)**	^**15**^ **N Recovery % Total**
**NO**_**3**_^**−**^	Arabidopsis	NO_0_	45 ± 1	19 ± 1	63 ± 1^*^
		NO_400_	36 ± 5	18 ± 1	**48 ± 7**
	Tomato	NO_0_	17 ± 1^*^	8 ± 1^*^	24 ± 2
		NO_400_	**14 ± 1**	**11 ± 1**	24 ± 1
**DON**	Arabidopsis	NO_0_	6 ± 1	3 ± 0	8 ± 1
		NO_400_	14 ± 6	2 ± 0	16 ± 6
	Tomato	NO_0_	1 ± 0	0 ± 0	2 ± 0
		NO_400_	2 ± 0	1 ± 0	2 ± 0
**MBN**	Arabidopsis	NO_0_	13 ± 3^*^	8 ± 1	24 ± 3
		NO_400_	**23 ± 3**	7 ± 2	30 ± 4
	Tomato	NO_0_	2 ± 1	2 ± 1	4 ± 1
		NO_400_	2 ± 0	2 ± 1	5 ± 1
**TN**	Arabidopsis	NO_0_	59 ± 3	19 ± 1	78 ± 4
		NO_400_	54 ± 5	18 ± 1	72 ± 6
	Tomato	NO_0_	24 ± 1^*^	11 ± 1	35 ± 0
		NO_400_	**21 ± 1**	12 ± 1	33 ± 2

## Discussion

Fumigation of plants with NO has allowed the identification of numerous NO-responsive genes and S-nitrosated proteins [46–50]. For example, this method shed light on the role of NO in regulating metabolism [51], nitrosoglutathion reductase activity [52] and salicylic acid (SA) biosynthesis [46]. Because NO can react with air to form NO_2_ that can also trigger stress responses in plants, caution is needed when interpreting results especially from NO fumigation experiments performed in closed chambers [53]. However, NO_2_ concentrations did not increase after NO flushing in the newly developed plant–soil mesocosm used in our study [25]. The NO concentration of 400 ppbv used in our experiment corresponds to natural soil fluxes [8, 9] and falls within the range reported for highly polluted urban environments (200–700 ppbv) [54].

We observed that the NO_400_ treatment of Arabidopsis and tomato did not impact most of the tested transcripts encoded by N-metabolism-, defense-, growth- and iron uptake-related genes. Nevertheless, NO_400_ significantly increased the expression of At*PAL1,* while decreasing that of At*PDF1.2* encoding phenylalanine ammonia lyase 1 and the plant defensin 1–2, respectively. These results are in accordance with previous published data reporting that NO promotes At*PAL* expression in Arabidopsis and tobacco [48, 55]. Regarding At*PDF1.2*, this jasmonate (JA)-responsive gene was found be regulated through NO-dependent processes in few studies. For instance, Huang *et al.* [46] observed an accumulation of At*PDF1.2* transcript in response to NO in plants impaired in SA biosynthesis, suggesting that SA acts as a negative regulator of the NO-dependent up-regulation of the corresponding gene. A NO-dependent induction of At*PDF1.2* expression was also found in *Arabidopsis* embryos [56]. Authors of this study proposed a model in which NO promotes the synthesis of JA which, in turn, represses the expression of the transcription factor MYC2 involved in the down-regulation of At*PDF1.2* expression. More recently, Pescador *et al.* [57] demonstrated that in Arabidopsis plants pre-exposed to volatiles promoting induced systemic resistance, the induced expression of At*PDF1.2* triggered by the pathogenic fungus *Botrytis cinerea* was inhibited by the NO scavenger cPTIO suppressing NO accumulation. Overall, our results strongly suggest that Arabidopsis plants perceived, directly or indirectly, exogenous NO. This was supported by the significantly larger leaf area in plants exposed to NO_400_. Similarly, exogenous NO has been shown to promote plant growth, including leaf expansion [58]. However, in our assays no physiological or biomass changes were observed in tomato leaves subjected to NO_400_. Although no effects of elevated NO concentrations on the aboveground biomass of tomatoes were detected, it should be noted that the mesocosm incubation system only allowed for undisturbed plant development up to 25 days after germination due to space constraint. However, the tomato plants were still growing, as evidenced by the greater CO₂ consumption during the final phase. Since this space constraint affected both NO_0_ and NO_400_ treated plants (no significant NO effect during phase 6), it cannot be considered a confounding factor and does not affect our conclusions. Nevertheless, our mesocosms provide a significant advance in the study of the role of NO on processes in the plant–soil systems. Future experiments using mesocosms optimized for plant development are needed to support and extend our results.

As previously reported [59, 60], we found that microbial communities were mostly influenced by the plant compartment, with the lowest alpha diversity in the root tissues, regardless of the plant species. The plant compartment also contributed up to 69% and 70% of the variation in the structure of the bacterial and fungal communities, respectively. Changes in microbial communities from soil to the endosphere were attributed to both abiotic and biotic filtering, driven by significant differences in nutrient availability, oxygen levels, and pH between soils and roots, as well as the selective recruitment of microorganisms by plants [60–62]. The enrichment of Proteobacteria in Arabidopsis and tomato roots is consistent with previous studies, although this plant effect can be modulated by soil type and plant genotype [63–67]. In line with the inability of Arabidopsis to establish a functional arbuscular mycorrhizal symbiosis [68], we observed the specific recruitment of Glomerales in tomato roots only. Glomerales include arbuscular mycorrhizal fungi that form symbiotic associations with most terrestrial plants, including tomato [69].

Although the plant compartment exhibited the strongest effects, we found that exposure to NO, either alone or in interaction with the plant compartment, also had a significant influence only on the diversity and the composition of the fungal community in the Arabidopsis experiment. The significant decrease in fungal diversity after NO_400_ exposure was concomitant with lower inorganic N pools, which may reflect the potential roles of fungi in organic matter decomposition and N turnover [70]. Since changes in NO homeostasis in Arabidopsis can affect microbial communities [66], this fungal response may have been indirectly mediated by NO-induced alterations in Arabidopsis physiology, even though this effect of NO was observed in the bulk soil. Thus, comparison of microbial communities between bulk (i.e. non-rhizospheric) and non-planted soils revealed significant effects of plants also in the bulk soil [71]. Accordingly, Schulz-Bohm *et al.* [72] showed that soil microorganisms can respond over long distances to volatile organic compounds emitted by plants, especially under stress. It is therefore not possible to decipher the mechanism by which NO influenced the fungal community, as it plays significant roles in various processes not only in plants but also across multiple fungal species, including spore formation, nitrogen metabolism, virulence and pathogenicity, stress tolerance capacity and hyphal extension [14, 73–76]. For example, NO can affect the balance between conidiation and sexual reproduction with higher NO levels both reducing conidiation and promoting cleistothecial formation in *Aspergillus* [75, 77]. Exogenous NO has been also associated with mycotoxin production in *Aspergillus* [75] and is related to plant infection in other fungal taxa such as *Botrytis* [76], *Blumeria* [78] or *Fusarium* [79]. While NO is known to have bactericidal effects through both oxidative and nitrosative stressors [15, 80], no significant effects of NO_400_ were observed on the diversity and composition of the bacterial community as previously observed in non-planted soil [16]. This could be due to the fact that bacteriostasis is the dominant manifestation of NO toxicity against most bacteria [81]. Alternatively, the NO concentration used in our experiment could have been too low to be toxic or that toxicity was limited to certain bacterial taxa, making such effect undetectable at the community level. Because of methodological constraints associated with the dynamic manipulation of NO concentrations, studies addressing the role of NO in soil communities or in association with plant roots are still scarce [16, 82]. Our study advances this field by applying an innovative mesocosm system to investigate how environmentally realistic NO concentrations shape bacterial and fungal communities in plant roots or in the rhizosphere.

Monitoring CO_2_ emissions revealed that the cumulative fluxes were negatively affected in the Arabidopsis experiment, whereas a significant decrease in total CO_2_ fluxes was observed only during phase 2 in the tomato experiment. The significant decrease in CO_2_ fluxes under elevated NO only during the photoperiod suggests a positive effect on photosynthesis with increased CO_2_ assimilation. In contrast, previous studies observed that exposure of lettuce to high NO concentration caused an inhibition of the net assimilation of CO_2_ due to a direct effect on photosynthesis rather than a change in stomatal conductance, and it did not affect respiration [83, 84]. However, in our study, the lower CO₂ fluxes with the light on, and higher fluxes with the light off, could be attributed to an overall increase in photosynthetic and respiratory activities due to the larger leaf area of Arabidopsis plants under NO_400_ treatment.

To further explore the impact of exogenous NO, we focused on N-cycling and quantified inorganic N-pools, N balance, N_2_O and NO emissions as well as the abundance of ammonia-oxidizers and denitrifiers. Along with lower ^15^N recovery of the labelled NH_4_^+^ as NO_3_^−^ under NO_400_ in the Arabidopsis experiment, we found lower abundances of AOB in the rhizosphere. This suggests a detrimental effect of NO_400_ on the nitrification process, which consists in the oxidation of NH_4_^+^ to NO_3_^−^ to generate energy for microbial growth. This decrease in the abundance of AOB, together with the reduced ^15^N recovery as NO_3_^−^, could partly explain the lower N_2_O fluxes observed under NO_400_ in the Arabidopsis experiment. Additionally, this detrimental effect on nitrifiers could further limit NO₃^−^ availability as a substrate for denitrification, ultimately leading to reduced N₂O emissions by denitrifiers. Since greater ammonium uptake by the microbial biomass was observed in the Arabidopsis experiment under NO_400_, this lower abundance of AOB may be due to increased competition for ammonium between assimilatory and dissimilatory microbial processes after exposure to NO_400_. Although earlier studies showed that the uptake of N by European beech roots increased significantly with elevated NO concentrations [85], the N-content in tomato plants was unaffected by NO_400_. This discrepancy can be explained by the fact that the NO effects on N uptake depend on soil N availability, with stronger effects observed under higher N availability [86]. Similarly, neither the abundance of N-cycling communities, N pools in soil, or analyzed plant transcripts showed clear trends in response to NO_400_ in the tomato experiment. As such, the observed reduction of up to 59% in N₂O fluxes—associated only with a slight increase in belowground biomass but not plant N content—remains unexplained and warrants further investigation. Previous studies showed that plant photosynthesis reduced N_2_O emission from plant–soil systems, without providing insight into the underlying mechanisms [87].

In conclusion, our experiments using an innovative plant–soil mesocosm system that dynamically changes background NO concentrations highlight that exogenous NO in soil can alter Arabidopsis physiology, accompanied by shifts in the fungal community and nitrogen cycling. Specifically, exposure to NO_400_ increased leaf area while reducing the abundance of certain N-cycling communities, ^15^N recovery as NO₃^−^, and cumulative CO₂ fluxes. Although most plant and microbial parameters remained unaffected in the tomato experiment, the substantial decrease in N₂O emissions following NO flushing is particularly intriguing, as it was not associated with detectable changes in microbial community composition, N pools, or the expression levels of the studied plant transcripts. Although the duration of the NO treatment in our experiment reflects continuous field NO measurements, potential long-term ecological effects may have been overlooked. Overall, our work opens intriguing perspectives on the potential contribution of exogenous NO to plant-microbe interactions but its broader impact on soil ecosystems appears limited and warrants further investigation through long-term experiments.

## Supplementary Material

Perez_Valera_NO_SUPPLEMENTARY_R1_ycaf237

## Data Availability

Raw sequence data of 16S rRNA and fungal ITS have been deposited in the Sequence Read Archive NCBI under BioProject accession numbers PRJNA1172607 and PRJNA1172642, respectively.
