# Supplementary material for: Assessing the role of exogenous NO on plants and microbial communities in soil
Source: ISME Commun. 2025 Dec 16;5(1):ycaf237. doi: 10.1093/ismeco/ycaf237 (PMC12766718; doi:10.1093/ismeco/ycaf237)
Supplement: Perez_Valera_NO_SUPPLEMENTARY_R1_ycaf237 [file perez_valera_no_supplementary_r1_ycaf237.pdf]

Assessing the role of exogenous NO on plants and microbial communities in soil

Eduardo Pérez-Valera, Logapragasan Subramaniam, Pauline Trapet, Antoine Berger, Marie-Christine Breuil, Florian Engelsberger, Nicolas Brüggemann, Klaus Butterbach-Bahl, Michael Dannenmann, David Wendehenne, Laurent Philippot

Supplementary Material

Table S1 2
Table S2 3
Table S3 4
Table S4 5
Table S5 7
Table S6 8

Figure S1. 9
Figure S2. 10
Figure S3. 11
Figure S4. 12
Figure S5. 13
Figure S6. 14

Supplementary Tables

Table S1. List of genes analysed and their function in Arabidopsis and tomato.

Plant	Gene	Function
Arabidopsis thaliana	*AtFER1*	Iron homeostasis
	AtAHB1, AtASN1, AtGLN1, AtGLN2, AtGLU1, AtGSNOR1, AtNIA1, AtNIA2, AtNIR1, AtNRT1, AtNRT2	Nitrogen metabolism
	AtBIN2, AtERF1, AtEXP8, AtHBI1, AtPAL1, AtPDF1.2, AtPR1, AtPR5, AtVSP2, AtWRKY33	Defense-growth balance
Solanum lycopersicum	*SlADH3, SlNR, SlPAL2, SlPhytoGB1*	Nitrogen metabolism
	SlARF5, SlARF7, SlEXPA5, SlGID1, SlGA20ox1, SlPR1, SlVSP2, SlXTH1	Defense-growth balance

Table S2. Primer sequences for quantitative RT-PCR analysis.

Plant	Gene	Gene code	Primer sequences, Forward (F) and Reverse (R)	
Arabidopsis	AHB1	At2g16060	F: TCTCGTTCTGATGGCTCCTGTG;	R: GTGTAACCGGTGATACTCTCGCC
	ASN1	At3g47340	F: GTGGCTTGTTCGACTGCAAAG;	R: TGAATCACAACTCCTTGACCCA
	EXP8	At2g40610	F: TGGTGCAATCCTCCTCTTCAGC;	R: TGGTACTCTTCGGAAAGAGACAGG
	BIN2	At4g18710	F : TGCACCCGAGCTCATATTTGGTG	R : TCTCCVVVAAATAATGGCTGACC
	ERF1	At4g17500	F: ATGGATCAAGGAGGTCGAGG	R: TCATTTCTTCTTGCCCTTGTTAGGTT
	FER1	At5g01600	F: TCGTTGAGAGTGAATTTCTGG;	R: ACCCCAACATTGGTCATCTG
	FRO2	At1g01580	F: CGATCGTTTCCTTCGGTTTC;	R: AATCCGAGCAGCGAGCAA
	GLN1	At5g37600	F: CATCCAAACCCTTGGTATTTGT;	R:GTAGCTGCGAAGGGTCAGTC
	GLN2	At5g35630	F GCAGCTTCTCCAACATGTCA	R : CCTGTCAGAGTAAGGTTTGGTG
	GLU1	At5g04140	F: AGTCGGGAACACTTGCTTGT;	R: TGTCATACCAGCAGCAACGT
	GSNOR1	At5g43940	F : GGTCTCTTTCCTTGTATTCTAG	R : GCATTCACGACACTCAGCTTG
	HBI1	At2g18300	F: CTGCCATATGGAACCTGTGGATAC;	R: GCTCTTCCCTTGTCTTAACCTCTG
	NIA1	At1g77760	F: ATCGTCAAAGAAACCGAAGTC;	R: ACGGAGCATGGATGAGTT
	NIA2	At1g37130	F: ACGGCGTGGTTCGTTCTTACA;	R: ACCTTCTTCGTCGGCGAGTTC
	NIR1	At2g15620	F: AGCGATTCCTCTTGATGC;	R: GTTCGTCGATAAGCCACA
	NRT1	At1g12110	F: ACACGCTCATGGTCCAACAG ;	R: AGATTAACGCTTCGCCGATACC
	NRT2	At1g08090	F: GCTTGCACGTTACCTGTGACC;	R: GCGTCCACCCTCTGACTTGG
	PAL1	At2g37040	F: AGATTAACGGGGCACACAAG;	R: AGTTGAGATCGCAGCCACTT
	PDF1.2	At5g44420	F: TTTGCTGCTTTCGACGCAC	R : CGCAAACCCCTGACCATG
	PR1	At2g14610	F: AAGGGTTCACAACCAGGCAC	R : CACTGCATGGGACCTACGC
	PR5	At1g75040	F: CGATAAGCCGGAAACTTGTC	R : AAGTGAAGGTGCTCGTTTCG
	VSP2	At5g24770	F: TCAGTGACCGTTGGAAGTTGTG;	R: GTTCGAACCATTAGGCTTCAATATG
	WRY33	At2g38470	F: CTTCCACTTGTTTCAGTCCCTCTC;	R: CTGTGGTTGGAGAAGCTAGAACG
	PTB	At3g01150	F: GATCTGAATGTTAAGGCTTTTAGCG;	R: GGCTTAGATCAGGAAGTGTATAGTCTCTG
	Expressed	At4g26410	F: GAGCTGAAGTGGCTTCCATGAC;	R: GGTCCGACATACCCATGATCC
Tomato	Actin	Solyc09g064370.2	F: CGGTGACCACTTTCCGATCT;	R: TCCTCACCGTCAGCCATTTT
	ADH3	Solyc09g064370.2	F: GGGAACAGTTGGTCTTGCTGTG;	R: ATGGGTTGCTCATGCTCCTTGG
	ARF5	Solyc04g081240.2	F: ATTAGTTCTGAGTTGTGGC;	R: GGTATCTGTGAAGTTGCTG
	ARF9	Solyc08g082630.2	F: CCAAGTTATCCTAATCTTCCTTCC;	R: GTAAAGCCTCCTGGTCATATTTG
	EXPA5	Solyc02g088100.2	F: AAGGGTTCAAGAACTCAATGGCAAC;	R: TTTGACGAACCCAACGAAGTCTCC
	GA20ox1	Solyc03g006880.2	F: CTCATTTCTAATGCTCATCGT;	R: TGCAGATGATTCTTTCTTAGCG
	GID1	Solyc09g074270.2	F: GATCTTGATACACCTCTCAGTACTA;	R: ACAGCCTT ACAT ATACTAACAAGAC
	NR	Solyc11g013810.3.1	F: AGTTCTCCCTCCACACGTTGAC;	R: TCTGGTTTGTACCACCAAGCTTCC
	PAL2	Solyc05g056170.2	F: AGGTGCTGAAATCGCTATGG;	R: TGTCAACTGCCTCCTCTGTCT
	PhytoGB1	Solyc07g008240.3.1	F: TGGAAAGGTTGTGGTGAGGGATTC;	R: TCAAAGTGCTCATCAACCACACC
	PR1	Solyc00g174340.1	F: TAGTCTGGCGCAACTCAGTC;	R: TGCAAGAAATGAACCACCAT
	LeVSP2	Solyc06g062390.2	F: CTGGTTATGCAGTCCCACAAT;	R: ACGTCGATATTGTTTGCCAAG
	TIP41	Solyc10g049850.1	F: ATGGAGTTTTTGAGTCTTCTGC;	R: GCTGCGTTTCTGGCTTAGG
	XTH1	Solyc03g031800.2	F: CTGCCACGCCACAAGAAGTCC;	R: TTTGACGAACCCAACGAAGTCTCC
	Expressed	Solyc07g025390.2	F: GCTAAGAACGCTGGACCTAATG;	R: TGGGTGTGCCTTTCTGAATG

Table S3. N content and biomass dry weight for tomato plants, distinguishing between aboveground (leaves and shoots) and belowground (roots) biomass across different NO treatment levels. Results are expressed as mean ± standard error (SE) based on six replicates. Statistically significant differences are marked with an asterisk (*p<0.05).

Plant	Biomass	Nitric oxide treatment	N content (mg)	Biomass (g)
**Tomato**	**Aboveground:** leaves and shoots	NO_0	242.9 ± 28.3	3.2 ± 0.3
		NO_{400}	277.0 ± 10.6	3.6 ± 0.1
	**Belowground:** roots	NO_0	18.4 ± 2.8	**0.38 ± 0.02 ***
		NO_{400}	17.6 ± 1.5	0.44 ± 0.01
	**Total:** leaves, shoots and roots	NO_0	261.3 ± 29.9	3.6 ± 0.4
		NO_{400}	294.6 ± 10.8	4.0 ± 0.1

Table S4. ANOVA results from two models to test i) the effect of NO, soil depth, and their interaction (model 1) and ii) the effect of NO, compartment (soil, rhizosphere, and root), and their interaction (model 2) on microbial alpha diversity (16S rRNA and ITS), qPCR-based abundance of bacteria, fungi, ammonia-oxidizers and denitrifiers, and N pools (NO_3^- and NH_4^+). Statistical tests are run independently for each experiment (Arabidopsis or tomato) and variable. Significant variables are indicated in bold.

		**Model 1** (bulk soil)				**Model 2** (all compartments)			
			**Df**	**F value**	***p value***		**Df**	**F value**	***p value***
		NO	1	0.34	0.564	NO	1	0.78	0.382
**Alpha diversity (16S rRNA)** OTU richness	**Arabidopsis**	**Depth**	**1**	**38.1**	**<0.001**	**Compartment**	**2**	**122**	**<0.001**
		NO:Depth	1	0.83	0.373	NO:Compartment	2	1.12	0.337
		NO	1	2.75	0.113	NO	1	1.37	0.249
	**Tomato**	**Depth**	**1**	**36.0**	**<0.001**	**Compartment**	**2**	**204**	**<0.001**
		NO:Depth	1	0.02	0.878	NO:Compartment	2	0.33	0.721
		NO	1	1.39	0.253	NO	1	0.14	0.708
**Alpha diversity (16S rRNA)** Shannon diversity	**Arabidopsis**	**Depth**	**1**	**107**	**<0.001**	**Compartment**	**2**	**79.4**	**<0.001**
		NO:Depth	1	0.02	0.883	NO:Compartment	2	0.01	0.989
		NO	1	4.16	0.055	NO	1	1.28	0.265
	**Tomato**	**Depth**	**1**	**111**	**<0.001**	**Compartment**	**2**	**59.5**	**<0.001**
		NO:Depth	1	0.27	0.612	NO:Compartment	2	0.61	0.550
		NO	1	1.50	0.234	NO	1	0.04	0.846
**Alpha diversity (ITS)** OTU richness	**Arabidopsis**	Depth	1	1.47	0.240	**Compartment**	**2**	**805**	**<0.001**
		NO:Depth	1	0.27	0.607	**NO:Compartment**	**2**	**3.40**	**0.043**
		NO	1	0.24	0.626	NO	1	2.49	0.123
	**Tomato**	Depth	1	1.31	0.265	**Compartment**	**2**	**324**	**<0.001**
		NO:Depth	1	2.03	0.170	NO:Compartment	2	0.04	0.961
		NO	1	3.36	0.082	**NO**	**1**	**6.18**	**0.017**
**Alpha diversity (ITS)** Shannon diversity	**Arabidopsis**	Depth	1	0.24	0.628	**Compartment**	**2**	**613**	**<0.001**
		NO:Depth	1	0	0.997	NO:Compartment	2	1.19	0.314
		NO	1	0.88	0.358	NO	1	0.14	0.714
	**Tomato**	Depth	1	0.67	0.424	**Compartment**	**2**	**16**	**<0.001**
		NO:Depth	1	0.65	0.430	NO:Compartment	2	0.91	0.409
		NO	1	0.12	0.728	NO	1	0.29	0.596
**Abundance of *amoA*** AOA	**Arabidopsis**	**Depth**	**1**	**5.24**	**0.033**	**Compartment**	**2**	**73.8**	**<0.001**
		NO:Depth	1	0.03	0.872	NO:Compartment	2	0.72	0.404
		NO	1	0.43	0.518	NO	1	0.04	0.837
	**Tomato**	**Depth**	**1**	**18.5**	**<0.001**	**Compartment**	**2**	**8.33**	**0.007**
		NO:Depth	1	0.55	0.466	NO:Compartment	2	0.52	0.477
		NO	1	0.02	0.878	NO	1	0.30	0.589
**Abundance of *amoA*** AOB	**Arabidopsis**	**Depth**	**1**	**88.9**	**<0.001**	**Compartment**	**2**	**14.0**	**0.001**
		NO:Depth	1	0.56	0.464	NO:Compartment	2	1.40	0.246
		NO	1	0.57	0.461	NO	1	0.08	0.781
	**Tomato**	**Depth**	**1**	**134**	**<0.001**	Compartment	2	0.16	0.689
		NO:Depth	1	0.05	0.830	NO:Compartment	2	0.93	0.343
		NO	1	0.61	0.444	NO	1	2.45	0.128
**Abundance of *amoA*** comammox A	**Arabidopsis**	**Depth**	**1**	**6.02**	**0.023**	**Compartment**	**2**	**5.32**	**0.028**
		NO:Depth	1	0.27	0.606	NO:Compartment	2	1.21	0.279
		NO	1	0.41	0.531	NO	1	0.07	0.796
	**Tomato**	**Depth**	**1**	**11.5**	**0.003**	**Compartment**	**2**	**53.7**	**<0.001**
		NO:Depth	1	0.07	0.800	NO:Compartment	2	0.99	0.328
		NO	1	0.05	0.833	NO	1	3.47	0.072
**Abundance of *amoA*** comammox B	**Arabidopsis**	Depth	1	0.01	0.930	**Compartment**	**2**	**25.9**	**<0.001**
		NO:Depth	1	2.57	0.125	NO:Compartment	2	4.52	0.042
		NO	1	0.11	0.738	NO	1	0.25	0.621
	**Tomato**	**Depth**	**1**	**10.4**	**0.004**	**Compartment**	**2**	**32.4**	**<0.001**
		NO:Depth	1	1.69	0.208	NO:Compartment	2	0.38	0.541
		NO	1	1.63	0.217	NO	1	0.36	0.552
**Abundance of *nirK***	**Arabidopsis**	Depth	1	2.34	0.142	**Compartment**	**2**	**35.2**	**<0.001**
		NO:Depth	1	0.38	0.547	NO:Compartment	2	5.77	0.023
		NO	1	0.27	0.611	NO	1	0.35	0.558
	**Tomato**	**Depth**	**1**	**11.7**	**0.003**	**Compartment**	**2**	**24.5**	**<0.001**
		NO:Depth	1	0.17	0.680	NO:Compartment	2	0.16	0.691

**Abundance of *nirS***	**Arabidopsis**	NO	1	0.20	0.656	NO	1	0.25	0.624
		Depth	1	0.46	0.503	**Compartment**	**2**	**21.5**	**<0.001**
		NO:Depth	1	1.30	0.268	NO:Compartment	2	1.36	0.253
	**Tomato**	NO	1	0.98	0.333	NO	1	1.13	0.296
		**Depth**	**1**	**6.84**	**0.017**	**Compartment**	**2**	**18.7**	**<0.001**
		NO:Depth	1	0.53	0.475	NO:Compartment	2	0.03	0.868
**Abundance of *nosZI***	**Arabidopsis**	NO	1	0.002	0.966	NO	1	1.55	0.222
		Depth	1	0.34	0.568	**Compartment**	**2**	**53.3**	**<0.001**
		NO:Depth	1	0.62	0.441	NO:Compartment	2	1.75	0.195
	**Tomato**	NO	1	0.95	0.342	NO	1	0.15	0.697
		Depth	1	4.83	0.040	**Compartment**	**2**	**27.7**	**<0.001**
		NO:Depth	1	0.12	0.730	NO:Compartment	2	0.69	0.412
**Abundance of *nosZII***	**Arabidopsis**	NO	1	0.04	0.836	NO	1	0.85	0.364
		Depth	1	0.47	0.502	**Compartment**	**2**	**26.1**	**<0.001**
		NO:Depth	1	0.38	0.544	NO:Compartment	2	1.83	0.186
	**Tomato**	NO	1	0.07	0.788	NO	1	0.03	0.872
		**Depth**	**1**	**4.83**	**0.040**	Compartment	2	1.04	0.315
		NO:Depth	1	0.00	0.964	NO:Compartment	2	0.07	0.800
**Abundance of 16S rRNA**	**Arabidopsis**	NO	1	0.09	0.771	NO	1	0.04	0.847
		Depth	1	2.01	0.172	Compartment	2	11.8	0.002
		NO:Depth	1	0.02	0.877	NO:Compartment	2	0.28	0.598
	**Tomato**	NO	1	0.44	0.513	NO	1	0.56	0.458
		Depth	1	0.02	0.902	**Compartment**	**2**	**22.8**	**<0.001**
		NO:Depth	1	0.13	0.721	NO:Compartment	2	0.11	0.739
**Abundance of ITS**	**Arabidopsis**	NO	1	0.13	0.720	NO	1	1.13	0.295
		Depth	1	0.85	0.368	**Compartment**	**2**	**142**	**<0.001**
		NO:Depth	1	0.37	0.549	NO:Compartment	2	0.23	0.638
	**Tomato**	NO	1	1.18	0.291	NO	1	3.30	0.079
		Depth	1	2.34	0.143	**Compartment**	**2**	**262**	**<0.001**
		NO:Depth	1	1.22	0.283	NO:Compartment	2	0.12	0.727

**NO_3^-**	**Arabidopsis**	NO	1	0.03	0.868
		Depth	1	0.84	0.371
		**NO:Depth**	**1**	**11.2**	**0.003**
	**Tomato**	NO	1	0.08	0.783
		Depth	1	1.71	0.207
		NO:Depth	1	4.15	0.056
**NH_4^+**	**Arabidopsis**	**NO**	**1**	**25.2**	**<0.001**
		Depth	1	4.94	0.038
		NO:Depth	1	0.16	0.697
	**Tomato**	NO	1	0.97	0.336
		**Depth**	**1**	**140**	**<0.001**
		NO:Depth	1	1.65	0.213

Table S5. Pairwise PERMANOVA for NO_{400} and NO_0 in bacterial (16S rRNA) and fungal (ITS) communities. Significant results (p<0.05) are indicated in bold.

	Plant	Compartment	F	R^2	*p value*	*p adjusted*
**16S rRNA**	Arabidopsis	Soil 0-5 cm	1.053	0.095	0.213	0.239
		Soil 5-10 cm	1.022	0.093	0.387	0.417
		Rhizosphere	0.956	0.087	0.561	0.561
		Root	1.003	0.100	0.447	0.464
	Tomato	Soil 0-5 cm	1.047	0.095	0.283	0.317
		Soil 5-10 cm	1.005	0.091	0.486	0.523
		Rhizosphere	0.891	0.090	0.715	0.715
		Root	0.649	0.061	0.619	0.642
**ITS**	**Arabidopsis**	**Soil 0-5 cm**	**1.458**	**0.127**	**0.041**	**0.044**
		**Soil 5-10 cm**	**1.669**	**0.143**	**0.021**	**0.024**
		Rhizosphere	0.906	0.083	0.520	0.520
		Root	1.270	0.113	0.222	0.230
	Tomato	Soil 0-5 cm	0.960	0.090	0.462	0.498
		Soil 5-10 cm	0.940	0.090	0.400	0.448
		Rhizosphere	0.830	0.080	0.629	0.652
		Root	0.590	0.060	0.977	0.977

Table S6. ^{15}N recovery percentages for tomato plants, distinguishing between aboveground (leaves and shoots) and belowground (roots) biomass across different NO treatment levels. Results are expressed as mean ± standard error (SE) based on six replicates. Statistically significant differences are marked with an asterisk (*p<0.05).

Plant	Biomass	Nitric oxide treatment	^{15}N Recovery %
Tomato	**Aboveground:** leaves and shoots	NO_0	79.5 ± 8.1
		NO_{400}	83.5 ± 3.4
	**Belowground:** roots	NO_0	5.0 ± 1.2
		NO_{400}	5.1 ± 0.5
	**Total:** leaves, shoots and roots	NO_0	84.4 ± 8.1
		NO_{400}	88.7 ± 3.4

Supplementary Figures

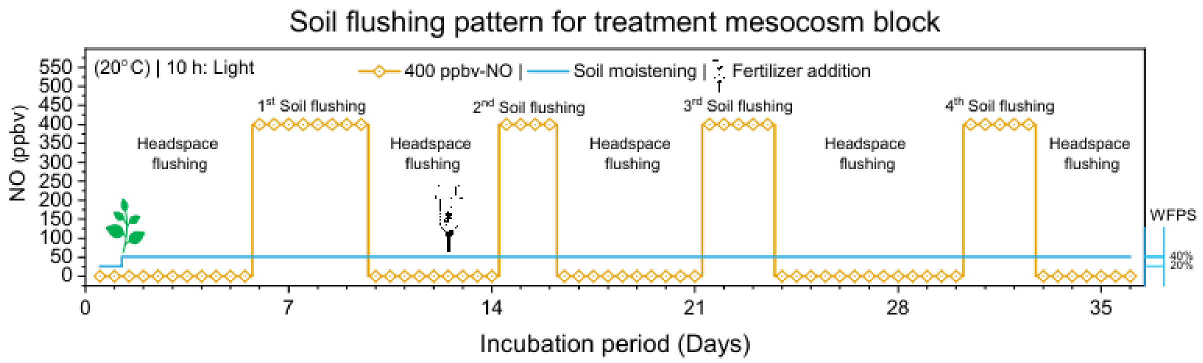

Figure S1. Diagram illustrating the experimental conditions for mesocosms treated with NO. The graph depicts the alternating flushing pattern, differentiating between headspace flushing (for trace gas flux measurements) and soil flushing (to establish a soil NO concentration of 400 ppbv). It also represents soil flushing patterns, water content adjustments, seedling planting, and fertilizer applications. Both treated (400 ppbv NO) and untreated (0 ppbv NO) mesocosms were randomly positioned within the thermostatic cabinet, with microcosm sampling conducted on day 37.

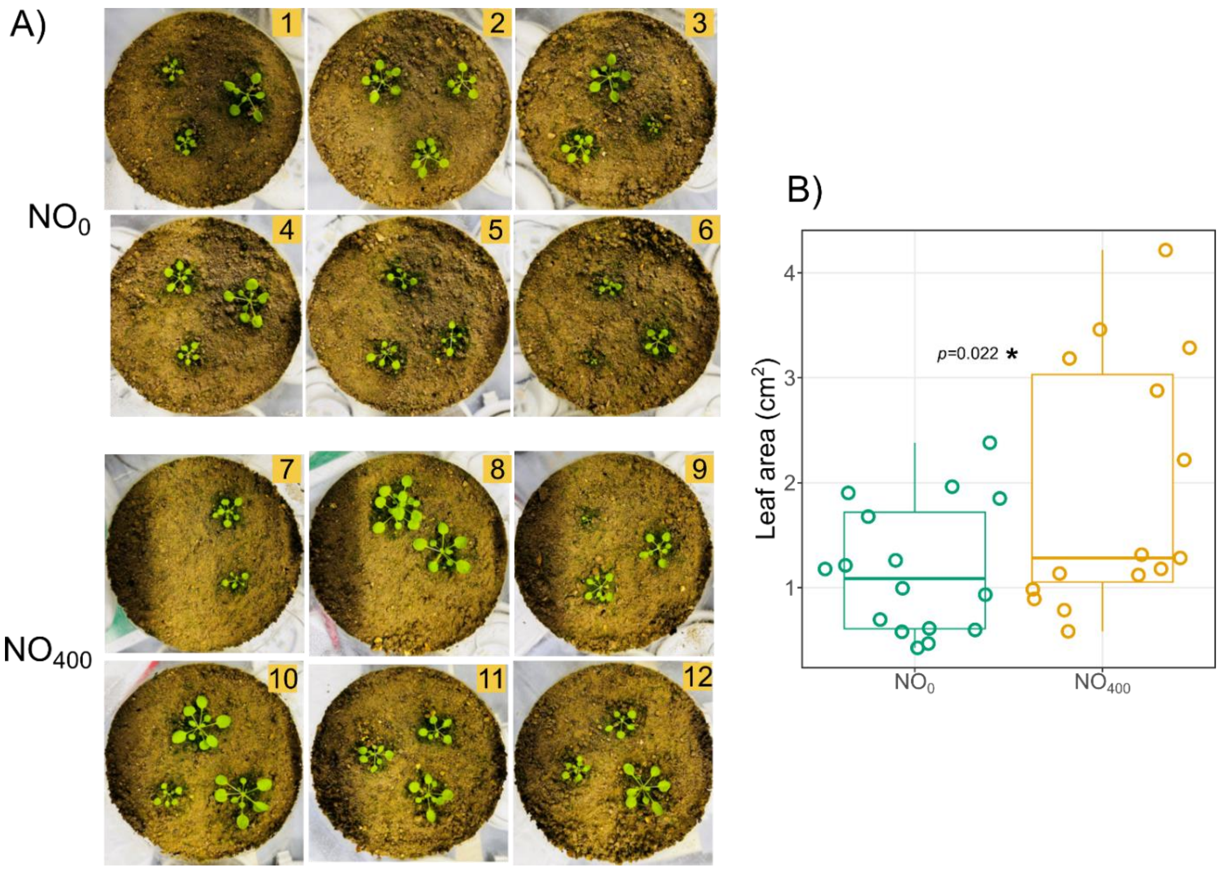

Figure S2. A) Growth of Arabidopsis in the mesocosms subjected to NO treatments (NO_0 and NO_{400}) over a 37-day incubation and B) boxplot showing the leaf area (in cm^2) of the Arabidopsis plants. Significant differences between NO_{400} and NO_0 were assessed with a t-test on log10-transformed data.

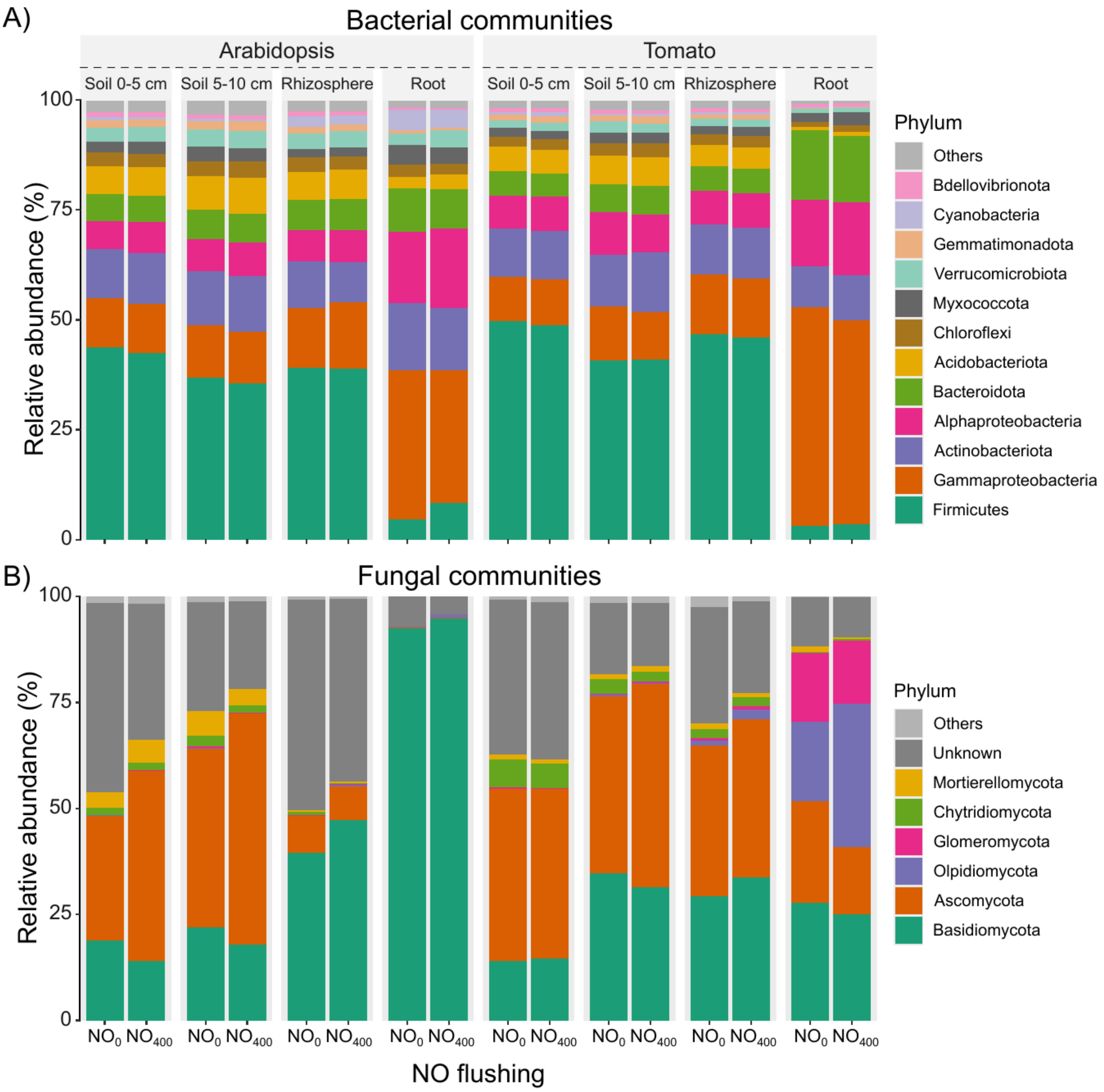

Figure S3. Barplots showing the relative abundance (in percentage) of the most abundant A) bacterial phyla (class for Proteobacteria) and B) fungal phyla in bulk soil, rhizosphere and root samples of Arabidopsis and tomato subjected to NO treatments (NO_0 and NO_{400}). The category others cluster taxa from less abundant phyla.

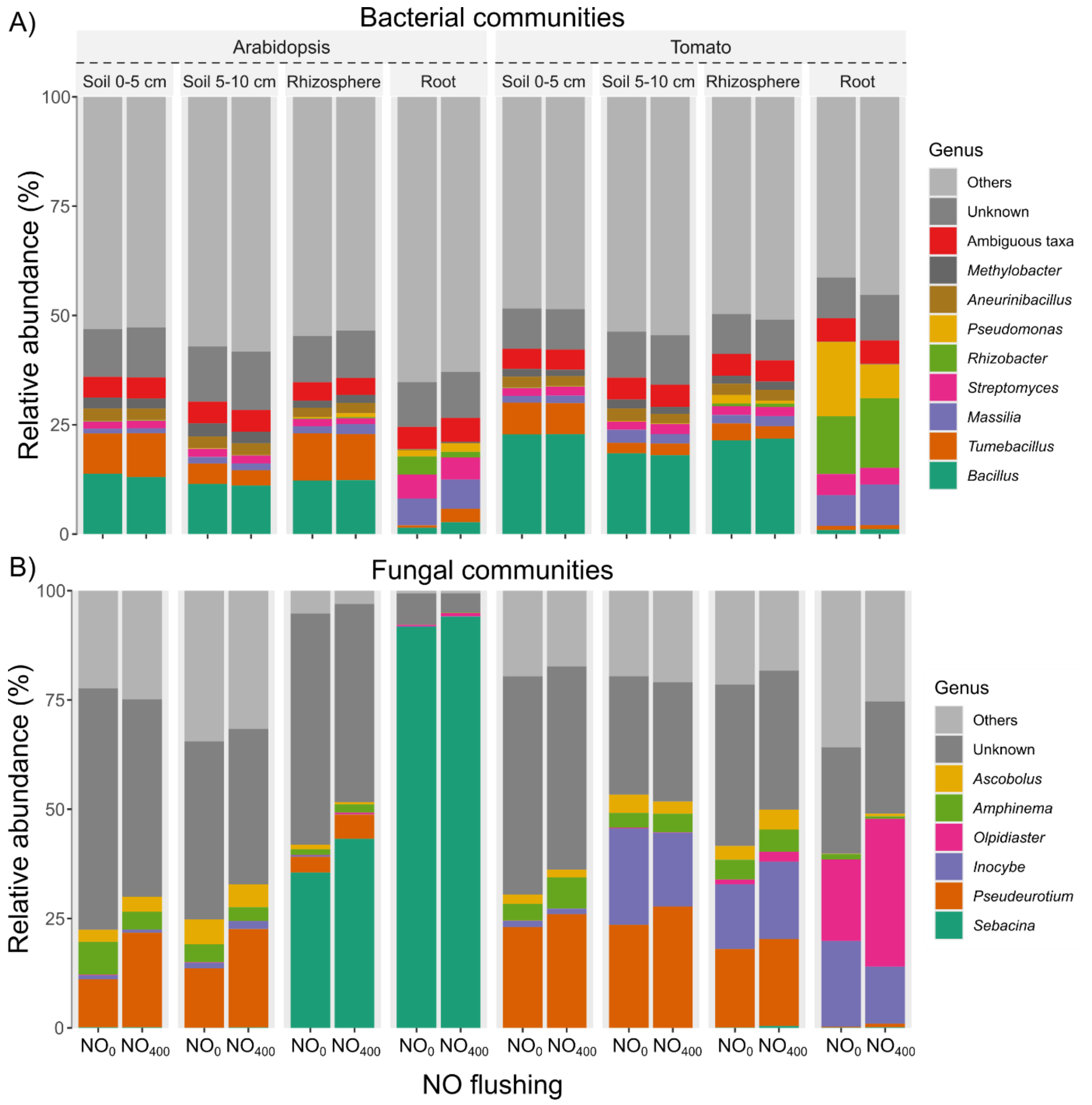

Figure S4. Barplots showing the relative abundance (in percentage) of the most abundant A) bacterial genera and B) fungal genera in bulk soil, rhizosphere and root samples of Arabidopsis and tomato subjected to NO treatments (NO_0 and NO_{400}). The category others cluster taxa from less abundant genera.

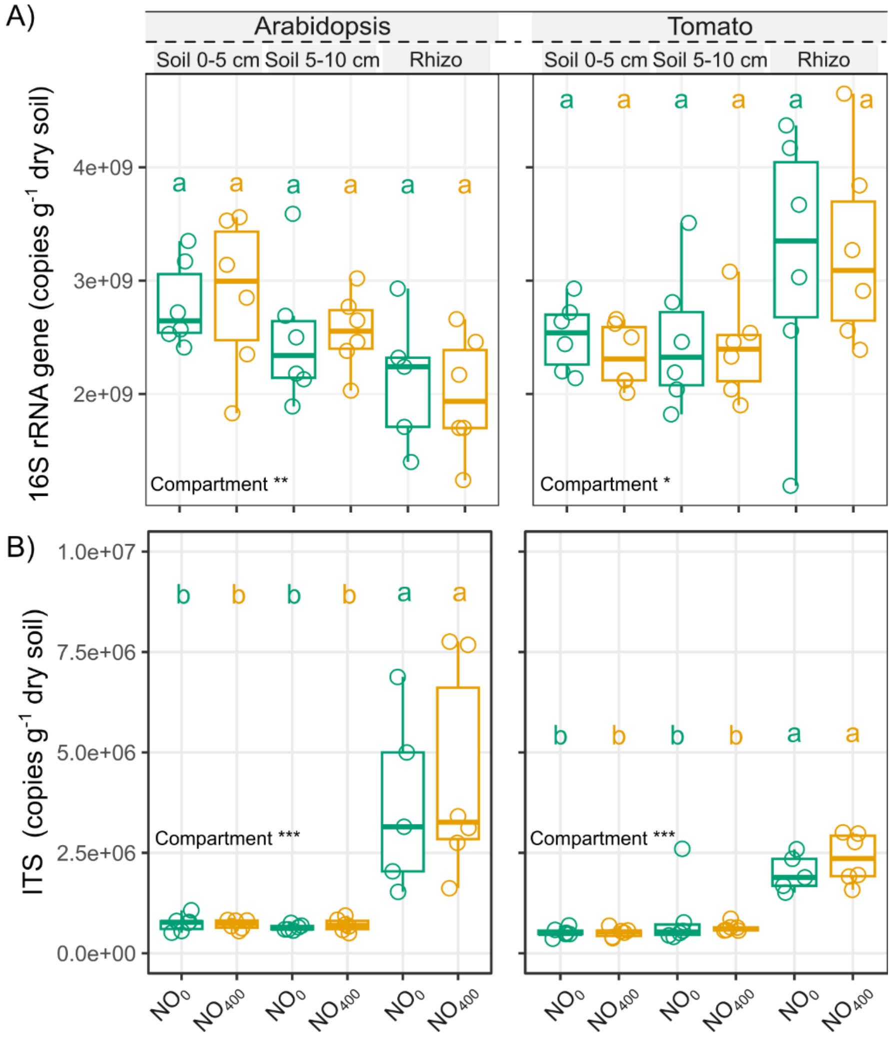

Figure S5. Boxplots showing the abundance of A) the total bacterial community based on the 16S rRNA gene copies and B) the total fungal community based on the ITS region in bulk soil and the rhizosphere of Arabidopsis and tomato plants subjected to NO_{400}. For each box, the central horizontal line indicates the median, while the lower and upper edges represent the first and third quartiles, respectively. The whiskers extend to the minimum and maximum values, provided they do not exceed 1.5 times the interquartile range. Distinct letters above the boxes indicate significant differences based on non-parametric Kruskal-Wallis tests, followed by Fisher's least significant difference with Benjamini-Hochberg correction for pairwise comparisons ($p<0.05$). Statistical significance for the effects of i) NO, soil depth, and their interaction, and ii) NO, compartment (bulk soil, rhizosphere and root), and their interaction, is indicated by asterisks in the figure (*$p<0.05$, **$p<0.01$, ***$p<0.001$), with model outputs provided in Table S4.

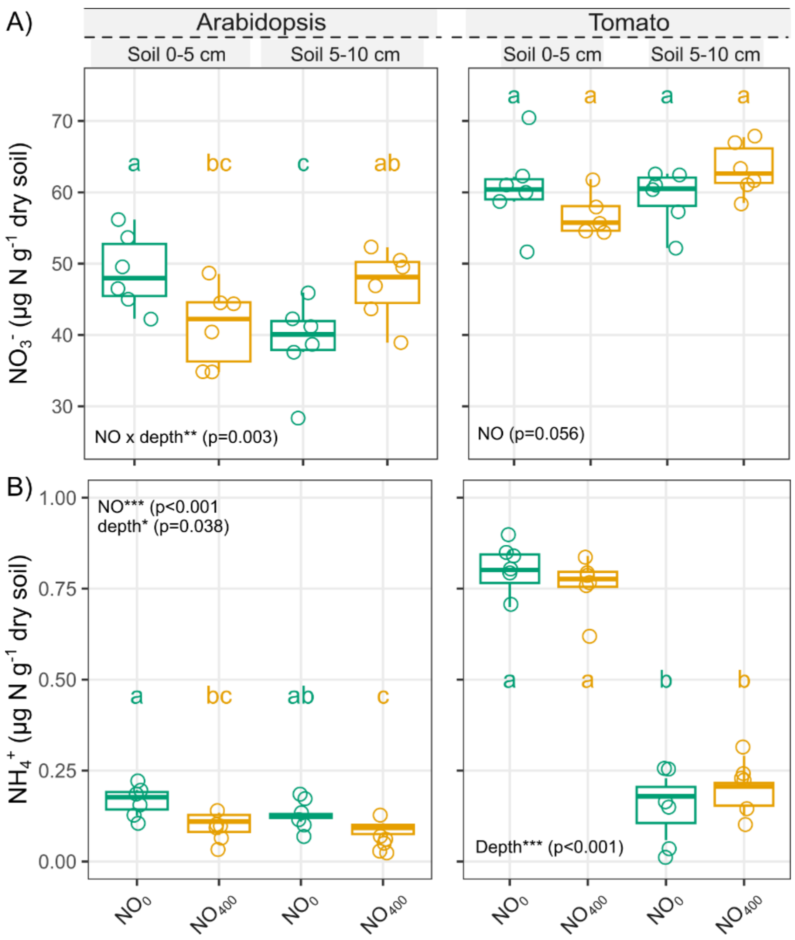

Figure S6. Boxplots showing the A) NO_3^- and B) NH_4^+ pools in bulk soil at 0-5 cm and 5-10 cm in mesocosms subjected to NO_{400}. For each box, the central horizontal line indicates the median, while the lower and upper edges represent the first and third quartiles, respectively. The whiskers extend to the minimum and maximum values, provided they do not exceed 1.5 times the interquartile range. Distinct letters above the boxes indicate significant differences based on non-parametric Kruskal-Wallis tests, followed by Fisher's least significant difference with Benjamini-Hochberg correction for pairwise comparisons (p<0.05). Statistical significance for the effects of i) NO, soil depth, and their interaction, and ii) NO, compartment (bulk soil, rhizosphere and root), and their interaction, is indicated by asterisks in the figure (*p<0.05, **p<0.01, ***p<0.001), with model outputs provided in Table S4.
